# Interleukin-6 promotes ferroptosis in bronchial epithelial cells by inducing reactive oxygen species-dependent lipid peroxidation and disrupting iron homeostasis

**DOI:** 10.1080/21655979.2021.1964158

**Published:** 2021-08-17

**Authors:** Fei Han, Shijie Li, Yankun Yang, Zhonghu Bai

**Affiliations:** a The Key Laboratory Of Industrial Biotechnology, Ministry Of Education, School Of Biotechnology, jiangnan university, Jiangsu, China; b National Engineering Laboratory For Cereal Fermentation Technology, Jiangnan University, Jiangsu, China; c Jiangsu Provincial Research Center For Bioactive Product Processing Technology, Jiangnan University, Wuxi, Jiangsu, China

**Keywords:** IL-6, asthma, ferroptosis, lipid peroxidation, iron homeostasis, bronchial epithelial cells

## Abstract

Asthma occurs accompanied by the ferroptosis in bronchial epithelial cells, during which Interleukin-6 (IL-6) plays a key role. However, the associations between IL-6, ferroptosis and asthma have not been reported. Bronchial epithelial cells BEAS-2B cells were induced by different concentrations of IL-6 and cell viability was detected by MTT assay. The TBARS production rate was detected by corresponding kit. The expression of oxidative stress-related indexes was detected by ELISA. The Iron Assay Kits detected total iron levels and ferrous ion (Fe^2+^) levels. Labile iron pool assay was used to detect the cell unstable iron pool. The expression of ferroptosis-related proteins was detected by Western blot. To further examine the mechanism of action, ferroptosis inhibitor Ferrostatin 1 (Fer-1), antioxidant NAC, and the iron supplement Fe were added. We found that IL-6 decreased the activity, promoted lipid peroxidation, disrupted iron homeostasis of BEAS-2B cells, and induced iron death in bronchial epithelial BEAS-2B cells. However, pretreatment with Ferrostatin-1 (Fer-1) and antioxidant NAC partially reversed the effect of IL-6 on lipid peroxidation and ferroptosis in BEAS-2B cells, while Fe augmented the effect. Overall, IL-6 promotes ferroptosis in bronchial epithelial cells by inducing reactive oxygen species (ROS)-dependent lipid peroxidation and disrupting iron homeostasis.

## Introduction

Asthma is a common chronic inflammatory airway disease that affects more than 300 million people worldwide [1]. Airway epithelial cells are the structural cells of the airway and the body’s first defense against inhaled stimuli, allergens, and pathogens [2]. Epithelial cells can synthesize and release a variety of inflammatory response mediators and cytokines, activate innate and adaptive immune cells, promote tissue regeneration and repair the damage triggered by environmental factors [3]. Studies have shown that defective repair of airway epithelial cells is one of the etiology of asthma [4,5]. The healing ability of epithelial cells in asthmatic patients was significantly lower than that in normal people [6]. Airway epithelial cells play an important role in the occurrence and development of chronic inflammatory diseases of the airway. It has become a hot spot of research on the pathogenesis and therapeutic target of chronic airway inflammatory diseases.

The important pro-inflammatory factor IL-6 induces the polarization of initial T cells to T helper cell 17 (Th17) cells and is in turn associated with neutrophil recruitment that can aggravate airway inflammation in asthma [7]. Recent evidence has suggested that the level of IL-6 in peripheral blood of patients with allergic asthma was increased significantly during the onset of the disease [8]. Michael C et al. [9] found that the expression of IL-6 in plasma of patients with asthma was significantly increased through cross-sectional study, suggesting that plasma IL-6 could be as a predictive biomarker for asthma. In addition, a significant association was found between serum IL-6 and asthma attack risk [10]. The IL-6 signaling pathway in dendritic cells plays a critical role in the uptake of allergens and the initiation of Th2/Th17 mediated airway inflammation and airway hyperresponsiveness in asthma, thus providing a new potential target for the treatment of allergic asthma [11]. Blocking the IL-6 pathway alleviates airway inflammation in asthmatic mice [12]. Thus, epithelial IL-6 signal transduction defines a novel asthma phenotype with increased airway inflammation [13].

Ferroptosis is a new type of programmed cell death characterized by intracellular iron accumulation and lipid peroxidation, which ultimately leads to cellular oxidative stress and cell death [14]. During ferroptosis, SLC7A11, a member of the solute carrier family, is down-regulated in ferroptosis cells and the deletion of SLC7A11 gene leads to lipid peroxidation and ferroptosis [15]. Intracellular iron homeostasis is mainly post transcriptional regulated by iron metabolism-related genes via iron response elements (iron regulatory protein system), such as ferritin (heavy-chain FTH1 and light chain FTL) [16]. Iron output is mediated by ferroportin 1 (FPN1, also known as SLC11A3), which releases Fe^2+^ into the cell and oxidize ferric to ferric [17]. At present, there are relatively few studies on the manifestations and mechanisms of ferroptosis related to asthma, but the existing studies suggest that ferroptosis plays an important role in the occurrence and development of asthma, especially in moderate to severe asthma. Inhibition of ferroptosis and oxidative stress contribute to the inhibitory effect of acupuncture on airway inflammation in an ovalbumin-induced mouse asthma model [18]. Induction of ferroptosis-like cell death exerts synergistic effects with glucocorticoids in allergic airway inflammation [19]. In addition, IL-6 causes ferroptosis of chondrocytes by inducing cellular oxidative stress and interfering with iron homeostasis [20]. IL-6 has an isozyme-specific effect on the expression of glutathione peroxidase (GPX). IL-6 can increase the GPX2 transcription concentration and decrease the GPX4 transcription concentration [21], while GPX plays a key role in the occurrence and development of ferroptosis [22]. However, the regulation of the relationship between IL-6 and ferroptosis in asthma has not been reported so far.

Our paper shows that IL-6 in asthma exacerbates asthma symptoms by inducing ferroptosis. Our paper can provide a solid theoretical basis for the clinical targeting of IL-6 in the treatment of asthma. In addition, in a clinical study, Brittany Esty et al. [23] treated two patients with severe persistent non-atopic asthma with tocilizumab, a human anti-IL-6 receptor (IL-6 R) monoclonal antibody. And the lung function of asthma patient improved and Th2 and Th17 effector cells decreased. However, at present, IL-6 McAb has not been widely used in the clinical treatment of asthma, and more clinical studies need to be reported. We have added the above to the discussion of the article.

Therefore, we hypothesized that IL-6 may play a role in asthma disease through the regulation of ferroptosis. In this paper, we discuss the role of IL-6 in asthma and its mechanism of action on ferroptosis of bronchial epithelial cells. Our paper lays a foundation for future research on the mechanism of ferroptosis in asthma.

## Materials and methods

### Cell culture model induction

Normal bronchial epithelial cells (BEAS-2B cells, BNCC254518) were obtained from the BeiNa Biological Technology Co., Ltd. Cells were cultured in DMEM (Gibco; Thermo Fisher Scientific, Inc.) with 10% FBS (Gibco; Thermo Fisher Scientific, Inc.) and 1% double resistant at 37℃ with CO_2_. Different concentrations of IL-6 (0, 5 ng/ml, 10 ng/ml, 25 ng/ml, 50 ng/ml) induced BEAS-2B cells for 24 h [20]. To further verify the effect of IL-6 on lipid peroxidation and ferroptosis in BEAS-2B cells, we added ferroptosis inhibitor Fer-1 (0.1 μM, CAS 347,174–05-4, Sigma–Aldrich, USA), iron supplement Fe (3.3 M, CAS 2238–05-8, Sigma–Aldrich, USA) [24] and antioxidant NAC (1 mM, CAS 616–91-1, Sigma–Aldrich, USA) [25]. The cells were divided into Control, IL-6, Fe+IL-6, Fer-1 + IL-6 and NAC+IL-6 groups.

### MTT(3-(4,5-Dimethylthiazol-2-yl)-2,5-diphenyltetrazolium bromide)

Cells were planted in 96-well plates at a density of 8 × 10^3^ cell/well. After corresponding treatment, 10 μL MTT solution was added into each well for 6 h, and the supernatant was discarded. Then 120 μL DMSO was used to detect the absorbance of each well at 490 nm with a microplate analyzer.

### Thiobarbituric acid reactive substances (TBARS) assay

Lipid peroxidation in the cells was estimated using the thiobarbituric acid-reactive substance (TBARS) assay [26]. 0.25 mL 15% (W/V) trichloroacetic acid and 7 μL of 500 mM butylated hydroxyanisole (BHA) were added to the cell lysate. The mixture were then centrifuged at 1000 g for 5 min. The supernatant was taken and 0.5 ml 0.375% (w/v) thiobarbituric acid was added. The mixture was then boiled for 10 min. After the mixture was cooled, TBARS was measured at 532 nm using a microplate reader (FilterMax F3/F5 Multi-Mode Microplate Reader, Molecular Devices).

### Lipid peroxidation assay

Glutathione peroxidase (GSH-px, S0073), Malondialdehyde (MDA, S0131M) and reactive oxygen species (ROS, S0033M) levels in BEAS-2B cells were measured using corresponding detection kits purchased form Beyotime Institute of Biotechnology (Shanghai, China) in accordance with the manufacturer’s instructions.

### Iron measurements

After the cells were treated by corresponding procedures, the total iron content (MAK025, Sigma–Aldrich) and Fe^2+^ content (AB83366, Abcam, UK) in the cells were detected by the corresponding kits according to the manufacturer’s protocol [27]. In brief, cells were added to an iron assay buffer, homogenized on ice, and then centrifuged at 13,000 × *g* for 10 min at 4°C, to obtain the supernatant for the assay. Finally, a 50-μL supernatant was incubated with 50 μL of assay buffer in a 96-well microplate for 30 min at room temperature. And then 50 μL of assay buffer was incubated with 200 μL of reagent mix in the dark for 30 min at room temperature. The microplate reader was used to measure the absorbance at 593 nm.

### Labile iron pool (LIP) assay

The LIP levels were measured using calcein-acetoxymethyl ester (calcein-AM, MedChemExpress, NJ, USA) and deferiprone (MedChemExpress), according to the methods described in the literature [28]. BEAS-2B cells were seeded in 12-well plates at a density of 6 × 10^5^ cells per well. After treated, cells were incubated with 0.05 μM Calcein AM for 15 min at 37°C. Then, cells were incubated with deferiprone for 1 h at 37°C. The medium containing deferiprone was replaced with fresh medium, and the cells were examined and imaged with a fluorescence microscope (BioTek Synergy 2).

### Western blot

BEAS-2B cells were collected and lysed with 0.5 μL RIPA lysis buffer (Beyotime Institute of Biotechnology) on ice for 30 min. Then, proteins were detected using a BCA protein assay kit (Bio-Rad Laboratories, Inc.). 30 µg of protein samples were separated by 10% SDS-PAGE and then transferred onto polyvinylidene fluoride (PVDF) membranes. After being blocked with 5% skimmed milk for 2 h at room temperature, the membranes were incubated with primary antibodies overnight at 4°C at 1:1000 dilution. On the next day, the membranes were incubated with horseradish peroxidase-conjugated secondary antibody (goat anti-rabbit IgG,1:5000, ab172130, Abcam). The signals were detected using enhanced chemiluminescence reagent (GE Healthcare) and Image J software (version 146; National Institutes of Health, Bethesda, MD, USA) was used to analyze the fold-changes of protein levels. The information of primary antibodies were as follows: anti-SLC7A11 (ab175186, Abcam, UK), anti- GPX4 (ab125066, Abcam, UK), anti- FTH1 (ab75972, Abcam, UK), anti- FPN1 (ab78066, Abcam, UK) and anti-GAPDH (ab8245, Abcam, UK).

### Statistical analysis

The data are presented as mean ± standard deviation (SD). A multigroup analysis was performed using one-way ANOVA, followed by Tukey’s post hoc analysis to compare the two groups. Statistical analysis was performed with Prism 5.0 (GraphPad Software, La Jolla, CA). *p* < 0.05 was considered statistically significant.

## Results

### IL-6 decreased the activity and promoted lipid peroxidation of BEAS-2B cells

**Figure 1.**
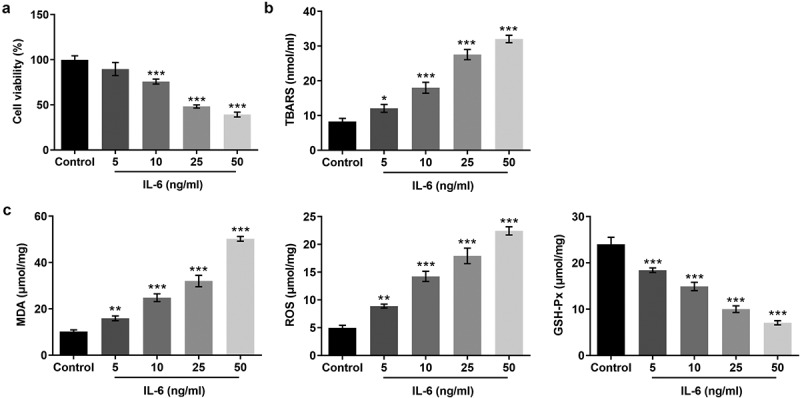
IL-6 decreased the activity and promoted lipid peroxidation of BEAS-2B cells. A. MTT assay detected the cell viability. B. The TBARS production rate was determined by the kit. C. The corresponding kits were used to detect the expression of the indexes related to oxidative stress. *p < 0.05, **p < 0.01, ***p < 0.001 vs Control

To detect the effect of IL-6 on BeAS-2B cells, MTT assay was used to detect the viability of BEAS-2B cells. The results showed that cell viability of these cells was decreased by IL-6 in a dose-dependent manner (Figure 1a). Subsequently, the TBARS production rate was detected by the kit to determine the cell lipid peroxidation level. The results showed that, compared with the control group, the expression of TBARS in cells induced by IL-6 was significantly increased, indicating that IL-6 can promote cell lipid peroxidation (Figure 1b). The levels of GSH-Px, MDA and ROS in cells were detected by the kits. We found that, compared with the control group, the expressions of MDA and ROS were significantly increased and the expression of GSH-Px was significantly decreased after IL-6 induction (Figure 1c). These results suggested that IL-6 decreased the activity and promoted lipid peroxidation of BEAS-2B cells.

### IL-6 disrupted iron homeostasis and induced ferroptosis of BEAS-2B cells

**Figure 2.**
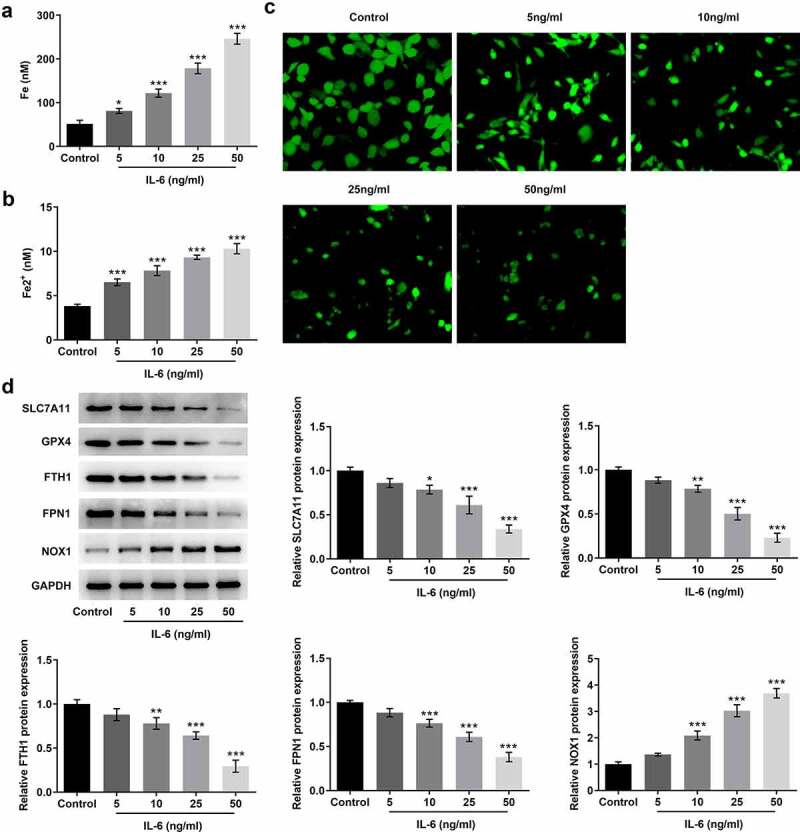
IL-6 disrupted iron homeostasis and induced ferroptosis of BEAS-2B cells. A. Iron Assay Kit detected total iron levels in cells. B. Iron Assay Kit detected Fe^2+^ levels in cells. C. LIP Assay was used to detect the iron expression. D. Western blot detected the expression of ferroptosis-related proteins. *p < 0.05, **p < 0.01, ***p < 0.001 vs Control

The iron kits were used to measure the levels of total iron and Fe^2+^ in cells. With the increase of IL-6-induced concentration, the levels of total iron and Fe^2+^ in cells were increased significantly (Figure 2a and b). The level of free iron can be determined by LIP Assay. The results showed that the LIP level was significantly decreased after IL-6 induction compared with the control group, indicating that the Fe^2+^ level in cells was increased (Figure 2c). This result is consistent with the previous expression trend of total iron and Fe^2+^. The expression of ferroptosis-related proteins was detected by Western blot. Compared with the control group, after IL-6 induction, the expressions of SLC7A11, GPX4, FTH1, and FPN1 were decreased while the expression of NOX1 was increased in a dose-dependent manner (Figure 2d). The induction trend of IL-6 was the most obvious at the concentration of 50 ng/ml, so we chose 50 ng/ml for subsequent experiments. These results showed that IL-6 disrupted iron homeostasis and induced ferroptosis of BEAS-2B cells.

### Pretreatment with Fer-1 and NAC partially reversed the effect of IL-6 on lipid peroxidation and ferroptosis in BEAS-2B cells, while Fe increased those effects

**Figure 3.**
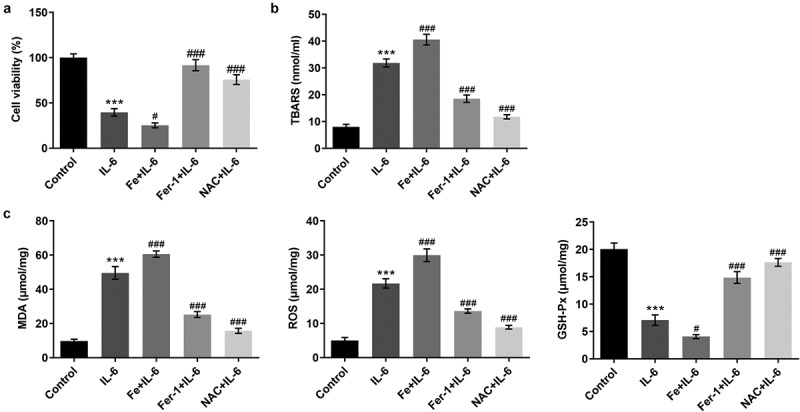
Pretreatment with Fer-1 and NAC partially reversed the effect of IL-6 on lipid peroxidation in BEAS-2B cells, while Fe increased those effects. A. MTT assay detected the cell viability after the induction of Fer-1, Fe and NAC. B. The TBARS production rate was determined by the kit after the induction of Fer-1, Fe and NAC. C. The corresponding kits were used to detect the expression of the indexes related to oxidative stress after the induction of Fer-1, Fe and NAC. ***p < 0.001 vs Control; #p < 0.05, ###p < 0.001 vs IL-6

**Figure 4.**
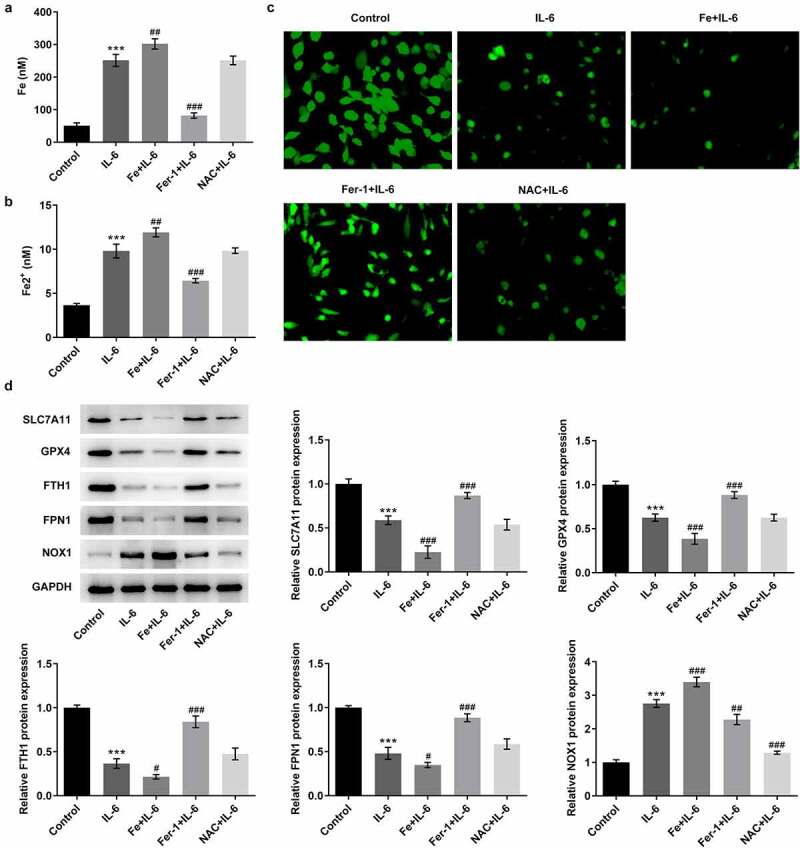
Pretreatment with Fer-1 and NAC partially reversed the effect of IL-6 on ferroptosis in BEAS-2B cells, while Fe increased those effects. A. Iron Assay Kit detected total iron levels in cells after the induction of Fer-1, Fe and NAC. B. Iron Assay Kit detected Fe^2+^ levels in cells after the induction of Fer-1, Fe and NAC. C. LIP Assay was used to detect the iron expression after the induction of Fer-1, Fe and NAC. D. Western blot detected the expression of ferroptosis-related proteins after the induction of Fer-1, Fe and NAC. ***p < 0.001 vs Control; #p < 0.05, ##p < 0.01, ###p < 0.001 vs IL-6

To further verify the effect of IL-6 on lipid peroxidation and ferroptosis in BEAS-2B cells, we added ferroptosis inhibitor Fer-1, iron supplement Fe, and antioxidant NAC. MTT results showed that compared with IL-6, the cell viability of Fe+IL-6 group was significantly decreased, while the cell viability of Fer-1 + IL-6 and NAC + IL-6 was significantly increased (Figure 3a). The results of lipid peroxidation showed that compared with IL-6, the lipid peroxidation level of Fe + IL-6 group was further increased, while the lipid peroxidation level of Fer-1 + IL-6 and NAC + IL-6 cells was reversed (Figure 3b). Compared with IL-6, the expression of MDA and ROS in Fe + IL-6 group was further increased, while the expression of GSH-Px was further decreased. The expressions of MDA, ROS and GSH-Px in Fer-1+ IL-6 and NAC+IL-6 cells presented opposite trends (Figure 3c). The iron homeostasis of the cells was then detected. The results showed that compared with the IL-6 group, the total iron and Fe^2+^ levels in the Fe+IL-6 group were increased, while the LIP level was further decreased. The levels of total iron and Fe^2+^ in Fer-1+ IL-6 group were decreased significantly, while the level of LIP was further increased. There were no significant change in total iron, Fe^2+^ and LIP levels in the NAC + IL-6 group (Figure 4a-c). Western blot results showed that compared with the IL-6 group, the expressions of SLC7A11, GPX4, FTH1, and FPN1 in Fe + IL-6 group were further decreased, while the expression of NOX1 was further increased. The expressions of SLC7A11, GPX4, FTH1, and FPN1 in Fer-1 + IL-6 group were significantly increased, while the expression of NOX1 was significantly decreased. There was no significant change in ferroptosis-related proteins in the NAC + IL-6 group (Figure 4d).

## Discussion

The normal bronchial epithelial cells act as a closed self-cleaning barrier in the body against inhaled allergens, pathogens, and other harmful substances [29]. Once damaged, they will release a large number of chemokines and cytokines, which can not only induce acute inflammatory response, but also regulate subsequent innate and adaptive immune responses [30]. Therefore, airway epithelial cells play an important role in asthma, which was why airway epithelial cells BEAS-2B were selected in this study for the experiments.

Ferroptosis is a type of cell death that plays an important role in iron homeostasis in asthma. In asthmatic airway epithelial cells, the interaction between ferroptosis and autophagy affect the pathological process of asthma, which may be used as a target for the treatment of asthma [31]. As accelerating eosinophilic death can effectively reduce allergic airway inflammation, the use of ferroptosis inducer that can potently complete this task may play a therapeutic role in allergic airway inflammation [19]. In an experimental model of asthma induced by house dust mite (HDM), the association between elevated lung iron levels and enhanced expression of TFR1 in airway tissue suggested that ferroptosis plays a role in the pathogenesis and severity of asthma and may be a potential therapeutic target [32]. A study published in Cell in 2017 showed a positive correlation between lipid peroxidation in airway epithelial cells and exhaled nitric oxide levels in asthmatic patients. Cigarette smoke, infection, and other factors that can lead to the decrease of GPX4 activity can cause the imbalance of lipid redox in epithelial cells, resulting in airway epithelial dysfunction and aggravating airway inflammation in asthma [33]. In the experiment, we detected the level of ferroptosis in BEAS-2B cells by measuring the level of lipid peroxidation and iron homeostasis.

IL-6 is a common marker of inflammation. However, recent studies suggested IL-6 as an inducer for pathogenesis of inflammatory diseases besides its widely known function as a pro-inflammatory marker [34]. IL-6 signaling pathway plays a key role in the control of differentiation and activation of T lymphocytes by inducing JAK/STAT-3 and Ras/ ERK/C/EBP pathways [35]. IL-6 can induce the polarization of primary T cells toward Th17 cells and promote neutrophil recruitment, thereby aggravating airway inflammation in asthma [11]. However, the specific mechanism has not been reported. Our experiment found that after IL-6 acted on bronchial epithelial cells, cell viability was decreased and lipid peroxidation occurred. In addition, IL-6 disrupted iron homeostasis and induced ferroptosis in BEAS-2B cells.

## Conclusion

Therefore, our experiment demonstrated that IL-6 promotes ferroptosis of bronchial epithelial cells BEAS-2B by inducing ROS-dependent lipid peroxidation and disrupting iron homeostasis. This study lays a foundation for the study on the role of IL-6 and ferroptosis in asthma disease.

### Limitations and future direction

There are some limitations in this article. IL-6 induces ferroptosis in BEAS-2B cells, and the expressions of SLC7A11, GPX4, FTH1, and FPN1 are significantly decreased. However, the specific regulatory mechanism has not been explored. In addition, our paper is a validation at the cellular level, but it has not been carried out on the animal level. Those experiments need to be further explored in our laboratory in the following experiments.

## Data Availability

The datasets analyzed during the current study are available from the corresponding author on reasonable request.

## Research highlights

IL-6 induces ROS-dependent lipid peroxidation and disruption of iron homeostasis

IL-6 induced ferroptosis in bronchial epithelial cells BEAS-2B

IL-6 exacerbates asthma by promoting ferroptosis in BEAS-2B cells
